# OD31 Value-Based Healthcare And Health Technology Assessment: Opportunities For Implementing A Colorectal Cancer Patient-Centered Care System

**DOI:** 10.1017/S0266462324001624

**Published:** 2025-01-07

**Authors:** Genevieve Asselin, Sylvain L’Espérance, Alice Nourissat, Marc Rhainds

## Abstract

**Introduction:**

The value-based healthcare (VBHC) concept links dollars spent to outcomes that matter to patients, rather than to the volume of services. A health technology assessment (HTA) was carried out to determine if the VBHC model could be effective to improve care delivery in colorectal cancer at the CHU de Québec-Université Laval.

**Methods:**

A systematic review on VBHC models implemented in oncology was conducted in indexed databases and grey literature between January 2000 and January 2022. Semistructured interviews were conducted from various key informants in our hospital (n=19), including three patients, to characterize the colorectal cancer care organization. HTA was conducted with an interdisciplinary group including surgeons, nurses, hospital managers, and patients.

**Results:**

Key factors to consider in the implementation of VBHC include organizing care around the same medical condition, measuring outcomes and costs for every patient, and tracking data through information technology platforms. Results from case studies suggest that VBHC may have promising effects on clinical, patient-reported, and process outcomes measures. Several issues regarding the degree of agreement with the VBHC principles have been identified in our facility, including a non-uniform care trajectory, a lack of an integrated interdisciplinary team, and the absence of efficient information technologies.

**Conclusions:**

It was recommended that the CHU de Québec-Université Laval initiates an organizational transformation in colorectal cancer care to implement a VBHC model. An effective care transformation will require a significant culture change with impacts on organization and practices but also with positive spin-offs by creating value and patient-centered care.

